# Monitoring of *In Vivo* Function of Superparamagnetic Iron Oxide Labelled Murine Dendritic Cells during Anti-Tumour Vaccination

**DOI:** 10.1371/journal.pone.0019662

**Published:** 2011-05-27

**Authors:** Richard Tavaré, Pervinder Sagoo, Gopal Varama, Yakup Tanriver, Alice Warely, Sandra S. Diebold, Richard Southworth, Tobias Schaeffter, Robert I. Lechler, Reza Razavi, Giovanna Lombardi, Gregory E. D. Mullen

**Affiliations:** 1 Division of Imaging Sciences and Biomedical Engineering, Department of Imaging Chemistry and Biology, King's College London, St. Thomas' Hospital, London, United Kingdom; 2 MRC Centre for Transplantation, King's College London, Guy's Hospital, London, United Kingdom; 3 NIHR Biomedical Research Centre, Guy's and St Thomas' NHS Foundation Trust and King's College London, London, United Kingdom; 4 Centre for Ultrastructural Imaging, King's College London, Guy's Campus, London, United Kingdom; 5 Division of Immunology, Infection and Inflammatory Disease, Peter Gorer Department of Immunology, King's College London, Guy's Hospital, London, United Kingdom; University of Palermo, Italy

## Abstract

Dendritic cells (DCs) generated *in vitro* to present tumour antigens have been injected in cancer patients to boost *in vivo* anti-tumour immune responses. This approach to cancer immunotherapy has had limited success. For anti-tumour therapy, delivery and subsequent migration of DCs to lymph nodes leading to effective stimulation of effector T cells is thought to be essential. The ability to non-invasively monitor the fate of adoptively transferred DCs *in vivo* using magnetic resonance imaging (MRI) is an important clinical tool to correlate their *in vivo* behavior with response to treatment. Previous reports of superparamagnetic iron oxides (SPIOs) labelling of different cell types, including DCs, have indicated varying detrimental effects on cell viability, migration, differentiation and immune function. Here we describe an optimised labelling procedure using a short incubation time and low concentration of clinically used SPIO Endorem to successfully track murine DC migration *in vivo* using MRI in a mouse tumour model. First, intracellular labelling of bone marrow derived DCs was monitored *in vitro* using electron microscopy and MRI relaxometry. Second, the *in vitro* characterisation of SPIO labelled DCs demonstrated that viability, phenotype and functions were comparable to unlabelled DCs. Third, *ex vivo* SPIO labelled DCs, when injected subcutaneously, allowed for the longitudinal monitoring by MR imaging of their migration *in vivo*. Fourth, the SPIO DCs induced the proliferation of adoptively transferred CD4^+^ T cells but, most importantly, they primed cytotoxic CD8^+^ T cell responses to protect against a B16-Ova tumour challenge. Finally, using anatomical information from the MR images, the immigration of DCs was confirmed by the increase in lymph node size post-DC injection. These results demonstrate that the SPIO labelling protocol developed in this study is not detrimental for DC function *in vitro* and *in vivo* has potential clinical application in monitoring therapeutic DCs in patients with cancer.

## Introduction

The ability to non-invasively image adoptively transferred dendritic cell (DCs) *in vivo* during cellular immunotherapy could be used to assess the *in vivo* behavior of injected DCs and correlate it to the clinical response to therapy. In particular, the ability to quantify the number of injected DCs that have migrated from the injection site to the draining lymph nodes (LN) would be a valuable clinical tool. To date, however, there has been limited success of DC based immunotherapies and many questions remain with regard to appropriate sites of delivery, frequency of delivery, cell number, DC phenotype, and optimal antigen for the most robust immune stimulation or tolerance induction [1]. Molecular imaging may provide some of the answers to these questions by using superparamagnetic iron oxide (SPIO) labelled DCs (SPIO DCs). To date, studies of SPIO DCs *in vivo* have focused mostly on evaluating their migratory properties using MRI and evaluating cell surface markers that are required for afferent LN migration but not on SPIO DC *in vivo* function [2], [3], [4], [5], [6].

Initial proof of concept studies demonstrated that DCs could be labelled with SPIOs and then monitored *in vivo* over time by MRI [2]. The efficiency of SPIO labelling of DCs was further enhanced by using SPIOs conjugated to anti-CD11c antibodies, resulting in a 50-fold increase in SPIO labelling of DCs [2]. However, this intense loading with SPIOs, with 30 pg or higher of iron per cell, was shown to adversely affect DC viability and migration by Verdijk et al. [3]. The use of MRI for the *in vivo* analysis of SPIO labelled DCs has also led to the discovery that the receptors CCR7 [4] and RAGE [5] are required for DC migration. Furthermore, MRI has allowed the study of DC migration to draining LNs in a semi-quantitative manner, showing a correlation between hypointensity in draining LNs and number of DCs injected [6]. In a recent study, rapamycin treated tolerogenic DCs were used in a graft-vs-host disease (GVHD) model where recipient tolerogenic DCs prolonged survival and decreased GVHD score on day seven post-bone marrow transplantation [7]. Monitoring of DC migration in this GVHD model, using tolerogenic DCs labelled with SPIO, showed hypointensity via MRI in the cervical LN eight days after injection, demonstrating the application of this approach to *in vivo* study of DC function. However, this study did not perform a comparison of SPIO labelled and unlabelled DCs. Similarly the cell numbers that migrated to LNs after injection were not quantified using SPIO labelled DCs [7]. Finally, in a clinical study the use of MRI and SPIO labelled human DCs has demonstrated that only around 50% of the cases the DCs were correctly injected into the lymph node despite ultrasound guidance and showed subsequent migration to other local LNs, [8].

Altogether, investigations to date have not studied the effect of SPIO labelling on DC immunological function *in vivo* compared to their unlabelled counterparts. So far SPIO labelled and unlabelled DCs have been compared for their ability to initiate T cell proliferation *in vitro*. Baumjohann et al. demonstrated that the capacity of SPIO DCs to prime an antigen-specific syngeneic T cell response by *in vitro* re-challenge was reduced by ∼30% compared to unlabelled DCs [4]. The authors suggested that the antigen presentation capacity of SPIO DCs remained intact *in vivo* but that SPIO-loading may have *minor* influences on migration and uptake or processing of antigens [4]. In a more recent paper using a low SPIO labelling efficiency of ∼6 pg iron per cell, a 15% decrease in *ex vivo* T cell proliferation was demonstrated when compared to unlabelled DCs [6]. The decreased stimulatory capacity of SPIO DCs compared to unlabelled DCs was confirmed further by demonstrating a decrease in their endocytic function by using dextran-FITC or fluorescently labelled apoptotic cells [6]. The explanation offered by the authors was that the dextran coat on SPIOs may impede the uptake of other glycosylated antigens through endocytosis. Utilising GFP^+^ DCs, they also showed that the number of GFP^+^ cells in the draining LN was reduced when DCs were SPIO labelled. The same group has repeated this using micrometer-sized iron oxides (MPIOs) and reported similar results where MIPO labelling decreased DC migration *in vivo* [9].

In this study, SPIO DC labelling efficiency, intracellular SPIO distribution, viability, phenotype, and ability to induce T cell proliferation were assessed *in vitro* and compared to unlabelled DCs. MRI was applied to monitor SPIO DC migration *in vivo* to the popliteal LN. When monitoring the migration of DCs over time, both SPIO labelled or unlabelled caused a similar increase in popliteal LN volume. DCs were labelled with sufficient SPIO to induce contrast hypointensity via MR imaging without affecting their *in vivo* function as compared to unlabelled DCs. This was demonstrated by an *in vivo* proliferation assay and the ability of SPIO DCs to induce protection against an Ova-B16 melanoma tumour challenge.

## Materials and Methods

### Ethics statement

Animal studies were carried out in accordance with UK Research Councils' and Medical Research Charities' guidelines on Responsibility in the Use of Animals in Bioscience Research, under a UK Home Office license (PPL# 70/6473; Title: Mechanisms of immunological response to foreign antigens).

### Mice, culture media, reagents and antibodies

C57BL/6 (H2^b^) and CBA/Ca (H2^k^) mice were ordered from Harlan Olac (Bicester, UK). OT-II and DO11.10 were bred and maintained in the Biological Services Unit at King's College London. RPMI 1640 medium (Sigma, Poole, UK) supplemented with 5 mM L-Glut (Invitrogen, Paisley, UK), 100 U/mL penicillin (Invitrogen), 100 µg/mL streptomycin (Invitrogen), 10% FCS (Harlan Sera-Lab, Loughborough, UK), 1 mM Hepes (Invitrogen) and 0.05 mM mercaptoethanol (Invitrogen) is used for all *in vitro* assays. DC media also contains 5% (vol/vol) supernatant from a granulocyte macrophage colony-stimulating factor (GM-CSF) secreting transfected cell line [10]. For T cell purification and washing steps, RPMI 1640 medium supplemented with 2% fetal calf serum (FCS) was used. Flow Cytometry was performed using a FACSCalibur (BD Biosciences) and the following phycoerythrin (PE)-conjugated antibodies (Abs): MHC-class I (IK^b^), MHC-class II (IA^b^), CD80, CD86, CD40, CD11c, CD54 and CCR7, and allophycocyanin conjugated anti-CD4. All antibodies and isotype controls were used according to manufacturer's instructions (BD Biosciences).

### Generation and SPIO labelling of mature bone marrow derived dendritic cells

DCs where generated as previously described [10], [11]. Briefly, bone marrow was isolated and treated with red blood cell (RBC) lysis Ack Buffer (150 mM NH_4_Cl, 1 mM KHCO_3_, 0.1 mM Na_2_-EDTA). Cells were then treated with a mixture of rat anti-mouse hybridoma supernatants containing anti-H2-E^k,d^/A^b,d^ (M5/114.15.2, TIB-120; ATCC, Manassas, USA), anti-CD45R/B220 (RA3-3AI/6.1; ATCC), anti-CD4 (YTS191; Therapeutic Immunology Group, Oxford, UK) and anti-CD8 (YTS169; Therapeutic Immunology Group). After washing with PBS, cells were incubated with goat anti-rat IgG DynaBeads (Dynal,Oslo, Norway) before separation in a magnetic field for negative isolation. Cells are resuspended in complete media plus GM-CSF and plated on 24-well plates (Barloworld Scientific, Staffordshire, UK) in 1 mL fractions at 1 –1.5×10^6^ cells/mL. Complete media plus GM-CSF was changed on days 2 and 4, removing non-adherent cells. On Day 7 DCs were harvested, incubated with 1 µg/mL of LPS (Sigma, *E. Coli* 026:B6) for 4 h, re-plated at 1×10^6^ cells/mL in a 24-well plate and concurrently incubated with or without 100 µg/mL of SPIO. Cells were washed three times with PBS before use in subsequent assays. The clinically approved SPIO agent Endorem was purchased from Gubert, France.

### Iron Quantification of SPIO labelled DCs

The average iron quantity per cell was calculated using the previously described calorimetric ferrozine-based assay [12]. Briefly, 2×10^6^ cells were counted, pelleted and lysed at −80°C for 30 min. and then shaken at room temp for 2 h in 200 µL of 50 mM NaOH. 100 µL of lysate was added to 100 µL of 10 mM HCl and 100 µL of iron releasing agent (freshly mixed solution of equal volumes of 1.4 M HCl and 4.5% (w/v) KMnO_4_ (Merck) in dH_2_O) for 2 h at 60°C. Once cool, 30 µL of iron detection reagent was added (6.5 mM FerroZine, 6.5 mM Neocuproine, 2.5 M ammonium acetate, 1 M ascorbic acid) for 30 min. 280 µL was transferred to a 96-well plate and absorbance is read at 550 nm. All samples were run in triplicate and compared to known concentrations of ferric chloride.

### Electron microscopy

SPIO labelled and unlabelled DCs were fixed for 1 h in 2% gluteraldehyde in 0.1 M phosphate buffer (pH 7.2) and post-fixed in 1% aqueous osmium tetroxide. Samples were then dehydrated through a graded series of ethanol and embedded in TAAB resin. Sections 70 nm thick were cut using a Leica ultracut E ultra microtome (Leica, Milton Keynes, UK), mounted on 200 mesh Cu grids (Agar Scientific, Stanstead, UK), and stained with 1.5% uranyl acetetate in 50% ethanol and 0.15% lead citrate before viewing on a Tecnai T12 electron microscope (FEI, the Netherlands). Images were captured using a Gatan Bioscan 792 camera (Gatan, Abington Oxon, UK). Energy filtered transmission electron microscopy (EFTEM) is a transmission electron microscopy technique in which those electrons that have suffered specific energy loss due to interaction with the atomic nuclei of a given element are used to form an image of the distribution of that element. EFTEM was carried out using a G2 Sphera 200 kV electron microscope (FEI, the Netherlands) fitted with a LaB6 emitter. Sections were imaged in transmission mode. Maps of Fe distribution were obtained with a GIF 2002 imaging filter, using TIA software.

### Magnetic Resonance Imaging

For the *in vitro* study MR relaxometry measurements were performed on a 1.5 T clinical whole-body MR unit (Achieva; Philips Medical Systems, UK). In order to study the influence of the labelled cells on the MRI contrast, different concentrations of SPIO labelled cells were imaged by multi-gradient-echo MR-sequence. For this, SPIO labelled DCs were diluted in 2-fold dilutions from 1×10^6^ to 6.25×10^5^ cells/mL gelatin (Sigma) and aliquoted in glass tubes that were then added to a gelatin filled dish. Also, free SPIO in gelatin was diluted in 2-fold dilutions from 50 to 0.78 µg SPIO per mL.

For quantification of the labelling process R_2_' parameter maps were used to differentiate cell-bound SPIO from free SPIO were calculated using the relationship: R_2_'  =  R_2_^*^ - R_2_ [13]. For this, relaxation R_2_ and R_2_^*^ rates were measured using a multi- spin-echo and gradient-echo sequences respectively to give magnitude based images at increasing echo-times (TEs). Taking the data on a pixel-by-pixel basis and plotting against its TE, a mono-exponential decay function of the form M_o_.exp(-R_2_^(*)^.TE) was fit to give maps of the respective relaxation rates. The R_2_ map was thus subtracted from the map for the R_2_^*^ relaxation rate taken at a similar geometric position, to generate a map for R_2_'.

MRI for the *in vivo* imaging of DC migration to the lymph node was performed on a clinical whole-body 3 T scanner (Philips Achieva) utilizing a 47 mm Philips microscopy coil. Mice were imaged sequentially by MRI on Days 0, 1, 2, and 4. MRI data was acquired using a gradient echo sequence with: flip angle  = 25°; FOV  = 45×30×8 mm; matrix  = 296×292; slice thickness  = 0.5 mm; TE/TR  = 4.6 ms/15 ms. As methods for detection of SPIO labelled DCs gradient echo images at longer echo times were used. Furthermore, a positive contrast technique with susceptibility gradient mapping using original resolution (SUMO) allows the selective visualization by post-processing [14]. The technique also provides quantitative information by measurement of the susceptibility gradient, *G_s_*, from the SUMO parameter maps used for positive contrast.

### Flow cytometry analysis

All flow cytometry acquisition and analysis was performed on a Becton Dickinson FACSCalibur running CellQuest software (Becton Dickinson, Oxford, UK) and FlowJo (Oregon, USA). For phenotype surface staining, 5×10^5^ DCs were incubated according to manufacturer's instructions with various antibodies in FACS Buffer (PBS plus 1% (vol/vol) FCS and 0.01% (w/vol) sodium azide) for 30 min. at 4°C in the dark followed by two washes in FACS Buffer. For *in vivo* proliferation experiments, flow cytometry is described in the methodology of the assay.

### Dendritic cell viability assays

Viability assays were performed at 4 or 18 h of SPIO labelling at 50, 100, or 150 µg SPIO per mL culture media. DCs incubated with SPIO under various labelling conditions were harvested and counted after the labelling procedure in a haemocytometer in the presence of Trypan Blue (Sigma). For the MTT assay, DCs incubated with SPIO under various labelling conditions were harvested and re-plated in a flat-bottomed 96-well plate (Iwaki) at 1×10^5^ cells per 100 µL media in triplicate. 10 µL of 5 mg/mL 3-[4,5-dimethylthiasol- 2-yl]-2,5-diphenyltetrazolium bromide (MTT, Sigma) was added to each well and incubated at 37°C for 4 h. 100 µL of 10% SDS in 1 mM HCl was added to each well and incubated at 37°C overnight. The absorbance of each well was read in a BioTek spectrophotometer at 570 nm.

### CD4^+^ T cell isolation

Spleens and lymph nodes (LN) were homogenized over 70 µm cell strainers (Beckman Coulter) to obtain a single cell suspension that was treated with ACK buffer. The washed cells were then incubated with a mixture of rat anti-mouse hybridoma supernatants containing anti-H2-E^k,d^/A^b,d^, anti-CD45R/B220, and anti-CD8 followed by goat anti-rat IgG DynaBeads before separation in a magnetic field. Purity of negatively selected CD4^+^ population was >93% before immediate use in further assays.

### *In vitro* proliferation assays

All proliferation assays were done in triplicate in 96-well plates with a volume of 250 µL. For peptide pulse OT-II proliferation assays, C57BL/6 DCs that had been treated ± SPIO and with LPS were pulsed with Ova_322−332_ that binds MHC-class II (A^b^) at 0.1 µg/mL for 30 min. After peptide pulse, the DCs were irradiated (∼3000 rad) and plated at 5×10^4^ DCs/well. DCs were incubated with freshly isolated CD4^+^ T cells from OT-2 transgenic mice from the spleen and lymph nodes and were mixed at various DC:T cell concentrations. Proliferation was assessed by ^3^H-thymidine incorporation in the last 18 h of 4-day cultures. Mixed lymphocyte reactions (MLRs) were performed similarly with irradiated DCs incubated with freshly isolated CBA/Ca CD4^+^ T cells at various DC:T cell concentrations. Again, proliferation was assessed by ^3^H-thymidine incorporation in the last 18 h of 4-day cultures. For protein pulse assays, DCs were treated with 10 mg/mL Ova protein (Sigma) for 4 h before SPIO labelling. After irradiation, DCs were incubated with isolated CD4^+^ T cells from OT-II mice at various DC:T cell concentrations. Proliferation was assessed by ^3^H-thymidine incorporation in the last 18 h of 4-day cultures.

### *In vivo* proliferation assay

Freshly isolated OT-II CD4^+^ T cells were incubated with 1 µM CFSE in 10 mL PBS per 1×10^7^ cells for 5 min. at 37°C and checked by flow cytometry for effective staining. 4×10^6^ cells in PBS were i.v. injected on Day 6. On Day 7 of DC culture, 1×10^6^ mature Ova_322−332_ pulsed DCs labelled with or without SPIO were injected in PBS s.c. in the heel. Animals were culled on Day 11 and specified organs were harvested. 1.5×10^6^ cells isolated were stained with anti-CD4-APC and 1×10^6^ events were collected for analysis by flow cytometry. Percent original dividing cells was the percentage of cells from the parent population that divided assuming that no cells died during the experiments. It was calculated by: (the total number of parent cell that had divided)/(the calculated number of total parent cells)x100. The proliferation index (PI) was the average number of divisions that the dividing cells have undergone. It was calculated by dividing the total number of events by the calculated number of parent cells.

### Tumour vaccination

On Day 0, C57BL/6 mice were s.c. immunised with 1×10^6^ DCs pulsed with Ova_257−264_ (SIINFEKL), labelled with and without SPIOs. On Day 4, 1×10^6^ B16 melanoma tumour cells expressing Ovalbumin and GFP were i.v. injected. Lungs were then harvested on Day 18 and counted for tumour nodules until a limit of 250 nodules per lung.

### Histology

Live DCs were allowed to bind to glass coverslips (Hendley, Essex, UK), fixed with 4% paraformaldehyde (PFA) for 10 min. on ice, washed two times with PBS and one time with dH_2_O. Slides were then added to 2% (w/vol) potassium ferrocyanide (Sigma) in 1 M HCl for 20 min. at RT. After two washes in PBS, slides are counterstained for 5 min. with Nuclear Fast Red (1% (w/vol) NFRed with 5% aluminum sulfate; Sigma) and washed again with PBS then dH_2_O. Mounting solution (Dako) was added before the addition of coverslip and imaging using a Lecia Leitz DMRB research microscope and Micro-Publisher 3.3 RTV camera. Freshly isolated LNs were embedded in OCT and snap-frozen in liquid nitrogen and stored at -80°C until sectioning. Prussian blue staining was performed with 17 µm sections that were fixed in ice-cold acetone for 1 min. Sections were then mixed for 30 min. at 37°C in 2% (w/v) potassium ferrocyanide (Sigma) in 0.5 M HCl. After thorough washing in water, sections were counter stained in 0.1% (w/v) Nuclear Fast Red (Sigma) with 5% aluminum sulfate. Slides were then mounted with Dako fluorescent mounting media for immediate imaging using a Lecia Leitz DMRB research microscope and Micro-Publisher 3.3 RTV camera. Images were analyzed with Image-Pro Plus 7.0 from Media Cybernetics software and Photoshop.

### Statistics

A t-test was performed to determine if there was a significant difference in percentage growth of LNs, in vivo proliferation or gradient susceptibility between SPIO labelled DCs and unlabelled DC; *P*-values of <0.05 were considered significant. To test for a significant differences between the Control, SPIO DC, DC + OVA peptide and SPIO DC + OVA peptide in the tumour challenge experiment, a One-Way ANOVA was first performed; *P*-values of <0.05 were considered significant. If the One-Way ANOVA was significant, then a post hoc analysis was performed with Student–Newman–Keuls pairwise comparison; *P*-values of <0.05 were considered significant.

## Results

### DCs can be efficiently labelled with SPIOs while maintaining their viability

In order to identify the optimal conditions for SPIO labelling of DCs using the clinically approved SPIO Endorem, various iron concentrations (50, 100, and 150 µg/mL) and incubation times (4 to 18 h) were investigated. No adverse effects were observed for all labelling conditions using both trypan blue and MTT assays. Due to previous literature reports of decreased viability, phagocytic function, and mobility at higher concentrations of SPIO and longer incubation times, SPIO labelling was performed at 100 µg/mL of Endorem for 4 h at 37°C [3], [6].

**Figure 1:**
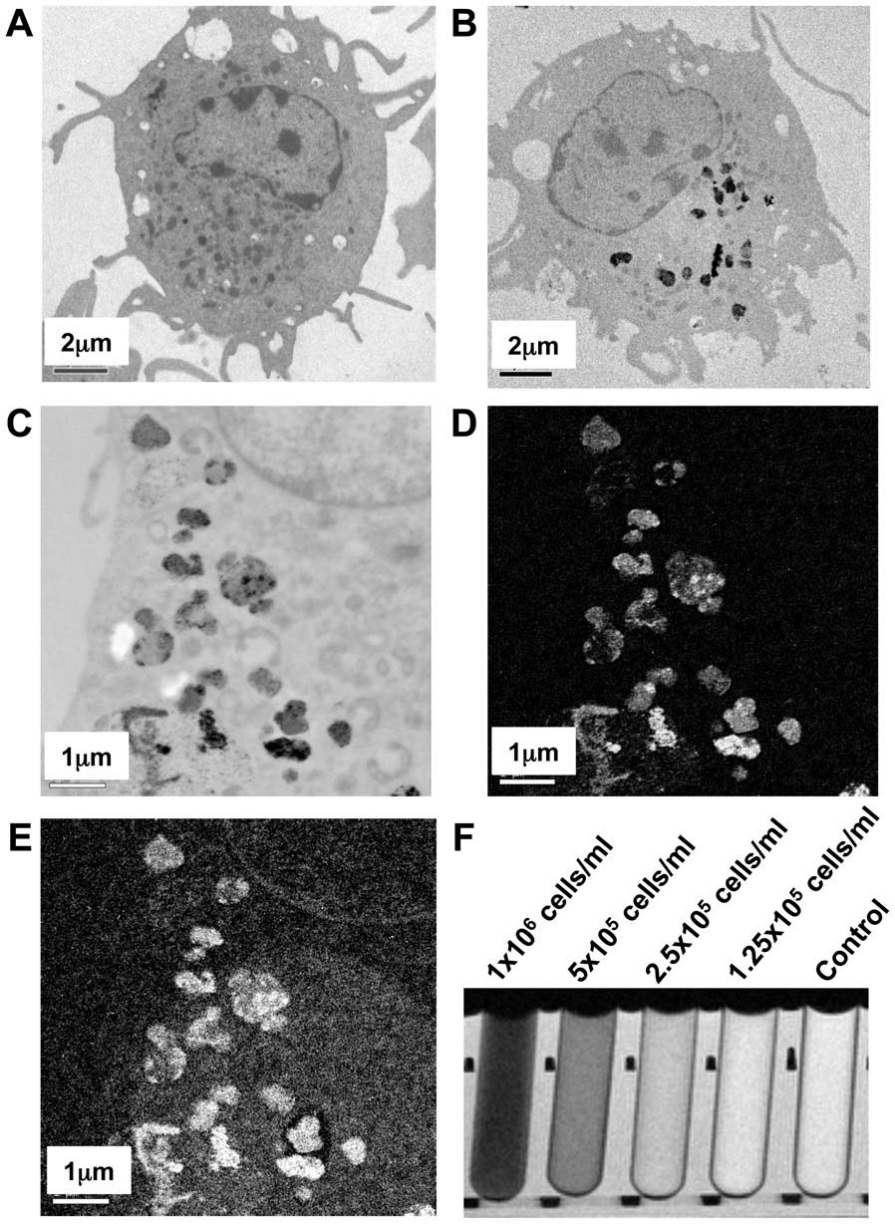
SPIO labelling of murine bone marrow derived DCs. Standard electron micrographs of **A**) unlabelled DCs, **B**) DCs incubated with 100 µg/mL of SPIO for 4 h at 37°C and **C**) enlarged region within a SPIO DC highlighting dark areas that contain iron oxide. Energy filtered transmission electron microscopy (EFTEM) of **D**) iron and **E**) oxygen. **F**) SPIO labelled cells were mixed in gelatin phantom at decreasing concentrations and imaged in a 1.5 T MRI with a T_2_* acquisition and a TE of 22.5 ms.

The average concentration of iron per cell under this labelling condition was determined by the ferrozine-based spectrophotometric iron quantification assay and showed an average of 7 pg iron per cell. The amount of iron in the cells was evaluated by using the Prussian blue stain (Figure S1). As reported before, the SPIO labelling was heterogeneous and some cells were heavily labelled while some not at all [4]. However, prussian blue staining revealed that >90% of DCs were stained blue for iron after SPIO labelling as seen by the presence of any blue stain in the DC under high magnification. Standard transmission electron microscopy (TEM) was performed to further analyse intracellular iron. Unlabelled cells did not show dark endosomal-like compartments as seen with SPIO labelled DCs (Figure 1A–C). To confirm that these dark regions within the DCs were SPIOs, energy filtered transmission electron microscopy (EFTEM) was used to map the iron and oxygen distribution within SPIO labelled DCs. Both the EFTEM analysis for oxygen and iron showed similar cellular localisation results (Figure 1D and E).

To ensure that the SPIO labelling condition changed the contrast in an MR image, SPIO DCs were set in gelatin at decreasing concentrations, imaged in a 1.5 T MRI scanner and compared to control unlabelled DCs (Figure 1F). At a TE of 22.5 ms, SPIO labelled DCs at 2.5×10^5^ cells/mL were more hypointense compared to control unlabelled DCs. Utilising R_2_, R_2_^*^ and R_2_' maps, intracellular iron uptake was confirmed in a phantom experiment (Figure S2A–C) [13]. The difference between R_2_ and R_2_^*^ mapping was used to determine R_2_', which increases in the presence of intracellular iron. The R_2_ and R_2_^*^ maps were used to show an increase in R_2_' values from SPIO DCs as compared to free SPIO in gelatin (Figure S2D).

### SPIO labelling did not affect the phenotype and *in vitro* function of DCs

The phenotype of SPIO DCs after maturation with LPS was analyzed by flow cytometry and compared to unlabelled DCs. Analysis of the common DC surface markers such as MHC-class I, MHC-class II, CD11c, CD40, CD80, CD86, CD54 and CCR7 showed that the phenotype of DCs with and without SPIO labelling was very similar (Figure S3).

**Figure 2:**
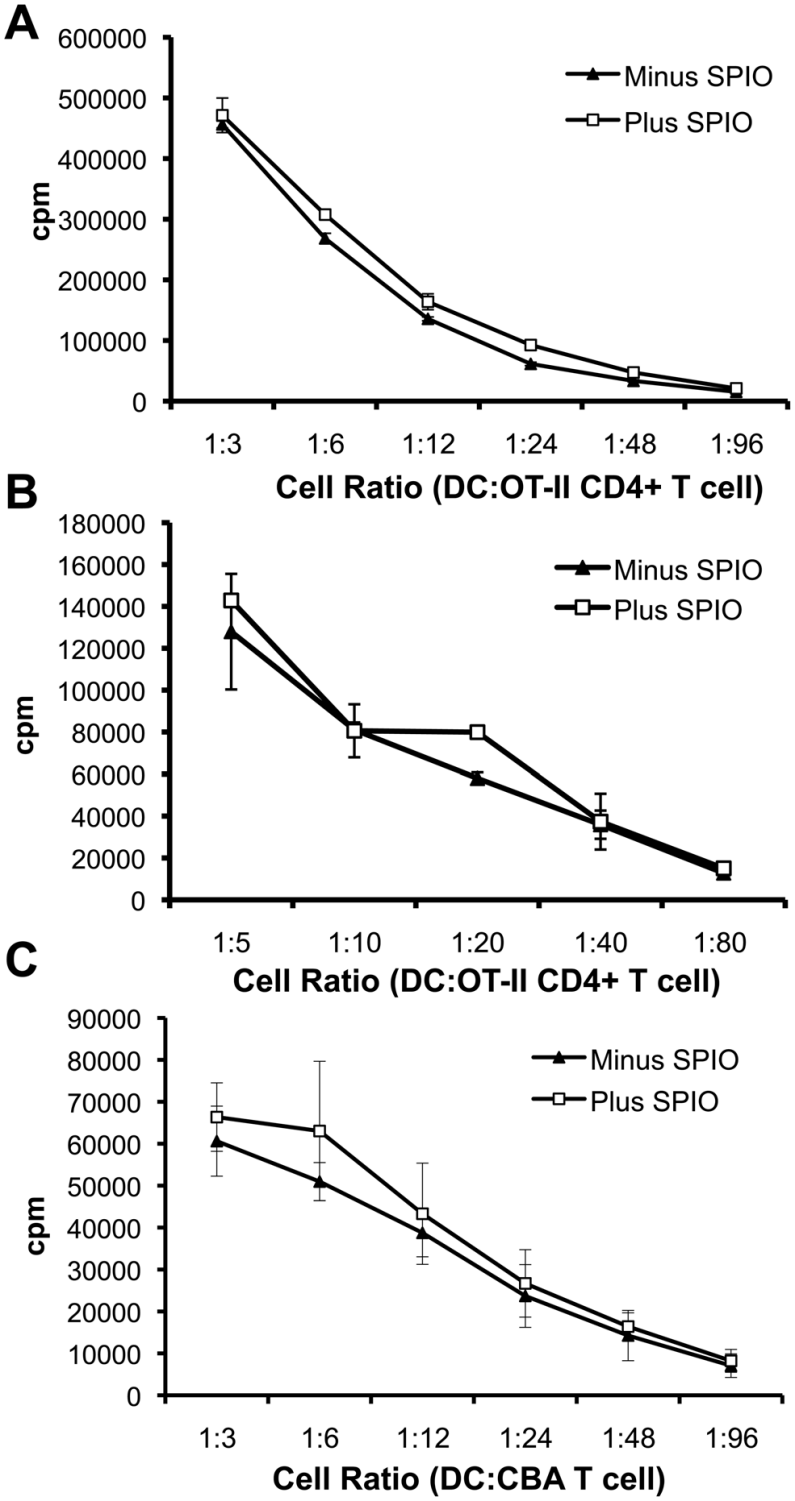
*In vitro* proliferation assays. **A**) SPIO labelled and unlabelled DCs (4 h at 100 µg/mL SPIO) were LPS treated and pulsed with Ova_322-332_ peptide for 30 min. Cells were then incubated with 5×10^5^ CD4^+^ T cells at varying DC dilutions. **B**) SPIO labelled and unlabelled DCs (4 h at 100 µg/mL SPIO) were LPS treated and pulsed with whole Ova protein for 30 min. Cells were then incubated with 5×10^5^ freshly isolated OT-II CD4^+^ T cells at various DC dilutions. **C**) SPIO labelled and unlabelled DCs (4 h at 100 µg/mL SPIO) were LPS treated and C57BL/6 DCs were incubated with 5×10^5^ freshly isolated CBA/Ca CD4^+^ T cells at various DC dilutions in a mixed lymphocyte reaction (MLR). In all *in vitro* experiments, cells were treated with tritiated thymidine on day three for 16 h to quantify proliferation. Experiments were repeated three times and data points are represented as the average of triplicate ± SE.

The functionality of SPIO DCs was analyzed firstly by assessing their T cell stimulatory capacity *in vitro*. SPIO labelled and unlabelled, LPS treated DC derived from BL/6 mice were pulsed with Ova_322−332_ peptide and then incubated with freshly isolated CD4^+^ T cells from OT-II mice (transgenic mice with a TCR specific for Ova, peptide 322–332, and restricted by H-2A^b^) at different DC:T cell ratios (Figure 2A). SPIO labelling of DCs did not influence OT-II CD4^+^ T cell proliferation. To test for the ability of SPIO DCs to retain efficient antigen processing and presentation functions, DCs were pulsed with the whole Ova protein for 4 h, prior to LPS maturation and SPIO labelling. DCs were then incubated with freshly isolated OT-II CD4^+^ T cells (Figure 2B). As presented in Figure 2B there was no change in the ability of SPIO DCs to stimulate antigen-specific CD4^+^ T cells. The functional consequence of SPIO labelling of DCs was also tested in MLR (Figure 2C). DCs derived from BL/6 mice were incubated with freshly isolated CD4^+^ T cells from CBA/Ca mice at different DC:T cell ratios. As shown in Figure 2C, SPIO labelling did not influence the stimulatory ability of DCs in a MLR.

### SPIO labelling of DCs did not influence their migratory properties

**Figure 3:**
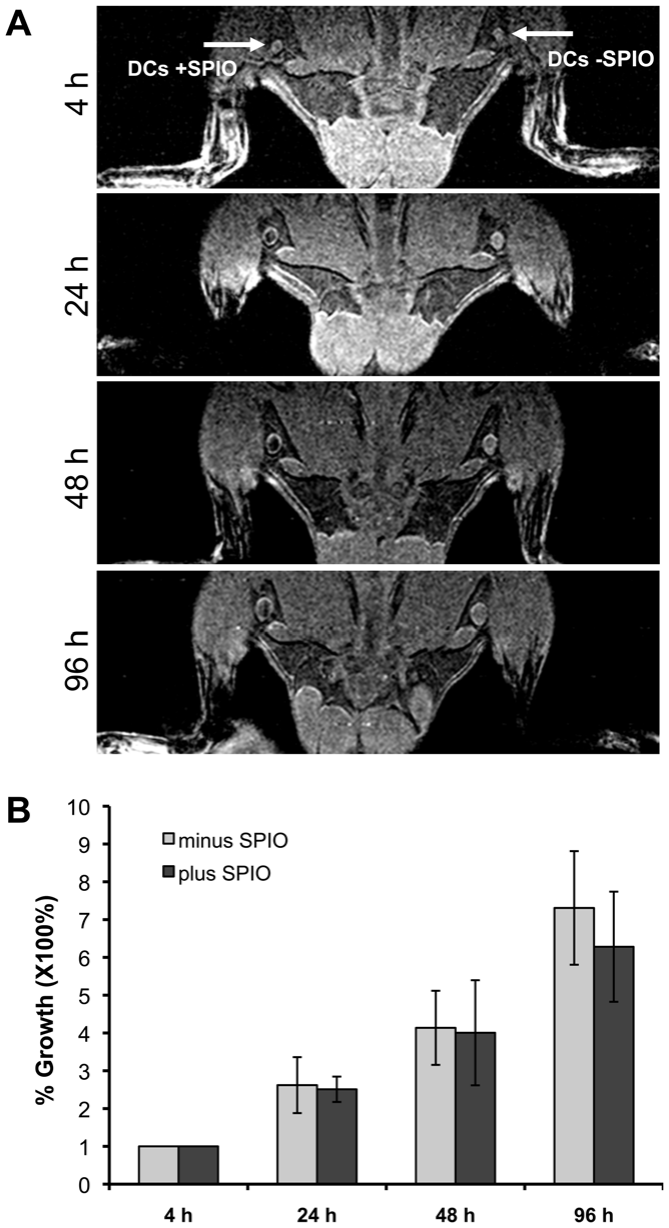
*In vivo* MR imaging of SPIO DCs. 1×10^6^ SPIO labelled or unlabelled DCs were injected s.c. in the right or left heel, respectively. **A**) The region encompassing the popliteal LNs was imaged serially over 96 h to observe the increase in size of both popliteal LN and detect hypointensity in the right popliteal LN. **B**) The growth of the popliteal LNs over time after injection of SPIO labelled or unlabelled DCs was analysed as the percentage growth in volume compared to the volume of the LN at 4 h post-injection. The percentage increase in LN volume was not significantly different between SPIO labelled and unlabelled DCs (P = 0.1888). Shown is a representative example of one experiment expressed as the average of triplicate ± SE.

To determine the effect of SPIO labelling on DC migration *in vivo*, mice were injected with 1×10^6^ SPIO DCs in the right leg and 1×10^6^ unlabelled DCs in the left leg. Mice were then imaged at 4, 24, 48, and 96 h post-DC injection (Figure 3A). This longitudinal imaging showed hypointensity as early as 24 h post-injection in the right popliteal LN as well as the original injection site. The hypointensity increased from 24 to 48 h post-DC injection. At 96 h, the hypointensity of iron in the right popliteal LN decreased. Furthermore, there was no significant difference in percentage LN increase between the right (SPIO labelled) and left popliteal LNs (unlabelled) (P = 0.1888). This indicated that the LNs grew similarly in size over time as a result of immune activation (Figure 3B). Indicating that *in vivo* there is no difference in migration and immune activation between unlabelled and SPIO labelled DCs.

Gradient echo MRI with longer echo times TE were used for visualization of iron in the popliteal LNs due to SPIO DC migration. MR Images of mice at 48 h post-DC injection with echo times of 4.6, 9.2, and 13.8 ms are shown in Figure S4A, B and C, respectively. The left popliteal LN, where unlabelled DCs were injected, showed no noticeable decrease in signal intensity with increase in TE. In fact, the LNs retain the “white” contrast while most of the signal was lost in the surrounding tissue. In the right LN, however, SPIO DCs caused a clear hypointensity present at 4.6 ms and increased at longer TEs.

**Figure 4:**
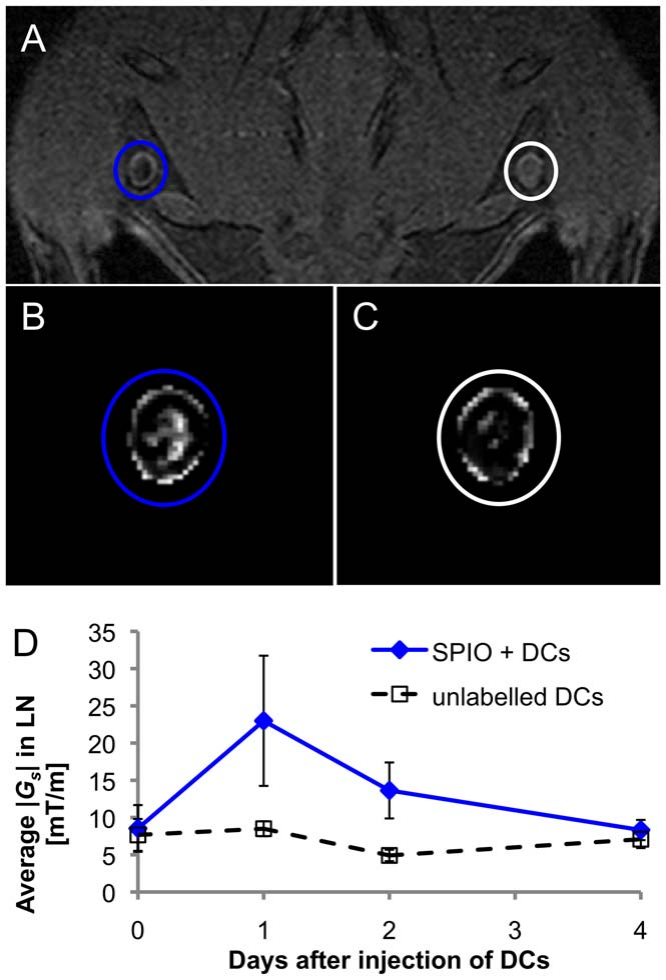
SPIO positive contrast in LN. 1×10^6^ SPIO labelled or unlabelled DCs were injected s.c. in the right or left heel, respectively. **A**) MR image at 48 h post-DC injection. Positive contrast images by SUMO at 48 h post-DC injection provide direct comparison of SPIO labelled and unlabelled DCs are compared in **B**) and **C**), respectively. **D**) Measurement of the susceptibility gradient, *G_s_*, in LNs from SUMO parameter maps over the longitudinal study indicates that *G_s_* was significantly higher for SPIO labelled DCs than unlabelled DC (P = 0.038).

As a second method for the detection of SPIO-labelled DCs the SUMO-technique was used for visualization with positive contrast (Figure 4). In both LNs there was positive contrast around the LNs due to changes in tissue density and susceptibility. In popliteal LNs that have SPIO DCs injected, there was a region of positive contrast in the centre of the LN (Figure 4B) whereas unlabelled DCs caused minimal positive contrast in the centre of the LN (Figure 4C). Furthermore, quantitative information over the longitudinal study was obtained by measurement of the susceptibility gradient, *G_s_*, from the SUMO parameter maps (Figure 4D) and *G_s_* was significantly higher for SPIO labelled DCs than unlabelled DC (P<0.038).

From the periphery, DCs enter afferent lymphatic vessels to migrate to and enter the LNs. Upon entrance to the LN they reside in the subcapsular sinus before migrating to the cortex of the LN where they present antigen to lymphocytes. To confirm the presence of iron in popliteal LNs after SPIO labelled DC were injected, as shown in the MRI images; prussian blue staining with a nuclear fast red counter-stain was performed on tissue cryo-sections (Figure S5). Popliteal LN samples taken 48 h post SPIO labelled DC injection reveals iron in both the subcapsular sinus and in the centre of the popliteal LN.

### SPIO DCs induced *in vivo* T cell proliferation and protected against tumour challenge

**Figure 5:**
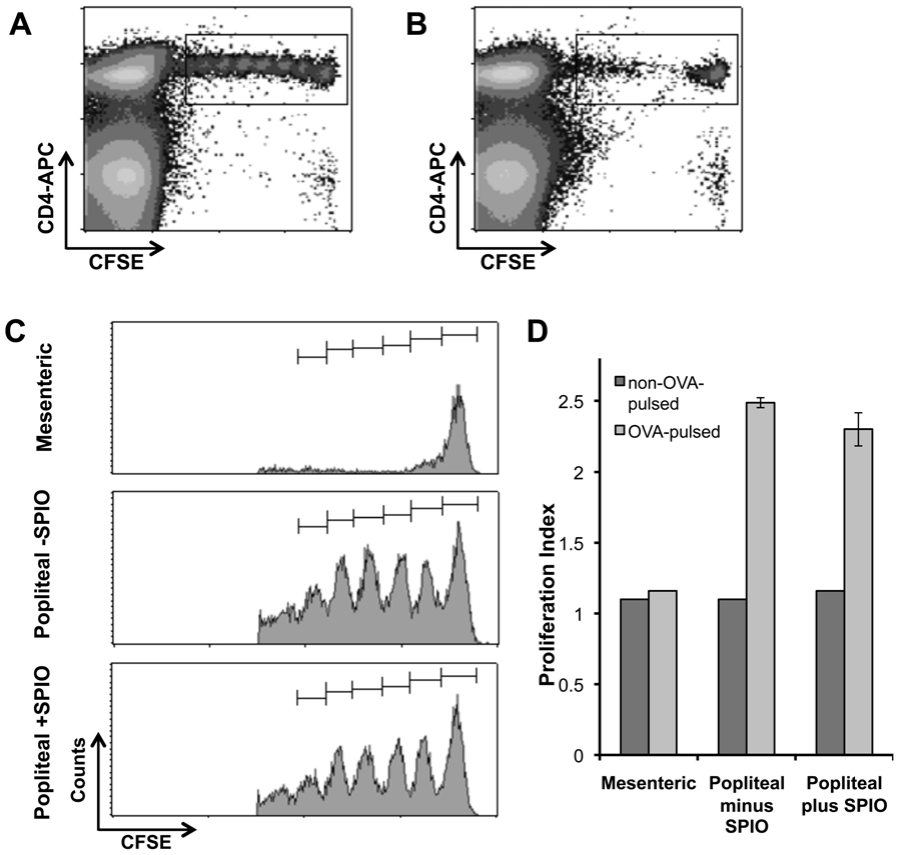
SPIO DCs induce *in vivo* T cell proliferation. 3×10^6^ CFSE labelled OT-II CD4^+^ T cells were injected i.v. The next day, 1×10^6^± SPIO, LPS treated, OVA_323-333_ pulsed DCs were injected s.c. heel. Four days later the mesenteric (**A**) and popliteal (**B**) LNs were harvested, stained with anti-CD4-APC and analysed by flow cytometry. (**C**) Regions of interest highlighted in (**A**) and (**B**) are shown as representative histograms and show the proliferation peaks of CFSE labelled OT-II CD4^+^ T cells in the control mesenteric LN (top panel), popliteal LN from mice which received unlabelled DCs (middle panel) and popliteal LN from mice receiving SPIO DCs (bottom panel). (**D**) Proliferation index ± SD after five divisions of CFSE labelled OT-II CD4^+^ T cells shows specific proliferation and no significant difference proliferation in popliteal LNs from mice which received DCs pulsed with OVA_323-333_ with and without SPIO (*P* = 0.061). These are representative examples of three individual experiments where non-pulsed DCs are run individually and pulsed DCs are run in triplicate.

If SPIO DCs are to be used to monitor adoptively transferred DCs in the clinic, then it is important to establish whether SPIO DCs can migrate and stimulate T cell proliferation *in vivo* in an equivalent manner to unlabelled DCs. To test this, mice initially received naïve CFSE^+^ OT-II CD4^+^ T cells followed 24 hours later by DC injection. Popliteal and mesenteric LNs were collected for individual analysis by flow cytometry 4 days after DC injections (Figure 5). When mature, Ova_322−332_ pulsed DCs were injected subcutaneously, proliferation was observed in the popliteal LN as seen by CFSE dilution of CFSE^+^ OT-II CD4^+^ T cells (Figure 5A). As expected, in the mesenteric LN little proliferation was observed (Figure 5B). Flow cytometry histograms of cells from three mice showed T cell proliferation in popliteal LNs when DCs were Ova_322−332_ pulsed and not mesenteric LNs (Figure 5C). As a control, no proliferation was seen when injected DCs were not pulsed with Ova_322−332_ peptide. The percentage of original CFSE^+^ OT-II CD4^+^ T cells dividing in the popliteal LN was 42.5 ± 1.3% and 40.1 ± 1.1% in unlabelled and labelled DC, respectively, while in the control mesenteric LN it was 9.9 ± 0.3% (data not shown). Similarly, the proliferation index (PI) of unlabelled and SPIO labelled DCs was 2.5 ± 0.04 and 2.3 ± 0.1, respectively, whereas the control mesenteric LN gave rise to a PI of 1.2 ± 2.1×10^−8^ (Figure 5D). Therefore, there was no significant difference between SPIO labelled and unlabelled DCs (P<0.061) in the ability to initiate T cell proliferation in the popliteal LN.

**Figure 6:**
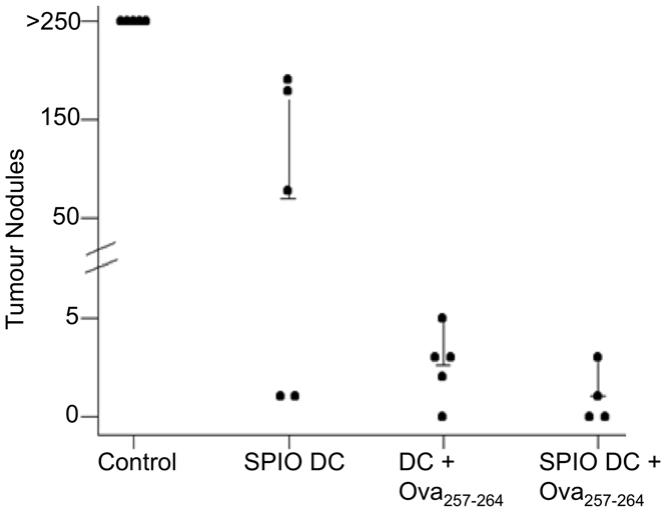
SPIO DC protect against B16 OVA tumour challenge. 1×10^6^ SPIO labelled or unlabelled DCs were injected s.c. in both the right and left heels 5 days before the i.v. injection of 1.5×10^6^ B16-Ova melanoma cells. 15 days later the lungs were harvested and tumour nodules counted with a limit of 250 nodules. A statistical significant difference in distribution of tumour nodule counts was found between all four groups (p<0.0001). Importantly, no significant difference in the efficacy of tumour vaccination was detected between SPIO DCs + Ova_257−264_ (labelled) and DCs + Ova_257−264_ (unlabelled) (P>0.05) and both were significantly different from control or SPIO DC (P<0.001). Shown is a representative example of three individual experiments expressed as the average + 95% confidence interval.

To further investigate whether SPIO labelling can affect DC function *in vivo* a tumour model was used. Mice were vaccinated in both heels with 1×10^6^ mature Ova_257−264_ pulsed DCs that were either SPIO labelled or unlabelled. Four days later, mice were challenged i.v. with 1.5×10^6^ B16-Ova melanoma cells. Fourteen days later, mice were sacrificed and surface lung nodules were counted (Figure 6). Mature, Ova_257−264_ pulsed, SPIO labelled and unlabelled DCs reduced the average number of tumour nodules to 1.0±1.4 and 3.2±1.9, respectively, per lung as compared to control mice with no therapy that had >250 nodules per lung (Figure 6). A statistical significant difference in distribution of tumour nodule counts was found between all four groups (p<0.0001). Importantly, no significant difference in the efficacy of tumour vaccination was detected between SPIO DCs + Ova_257−264_ (labelled) and DCs + Ova_257−264_ (unlabelled) (P>0.05) and both were significantly different from control or SPIO DC (P<0.001).

## Discussion

In this manuscript we have shown that SPIO labelling of DCs, using the conditions selected in this study, (i) does not affect the viability and phenotype of DCs; (ii) does not affect their stimulatory capacity *in vitro* and *in vivo*; (iii) allows the visualization of SPIO DC migration to the popliteal LNs over time and (iv) allows the visualization of DC contribution to anti-tumour responses. Altogether these results support the use of MRI to follow DCs injected *in vivo* for either the induction of an anti-tumour response or for their capacity, when “tolerogenic,” to prevent allograft rejection or autoimmune diseases.

Recent reports in the stem cell field, comparing SPIO labelling and reporter gene imaging of transplanted stem cells in the myocardium, have shown that while hypointensity due to SPIO is retained in the heart for up to 40 days, reporter gene imaging of the same cells is gone after 3 days [15], [16], [17]. SPIO was found to be localized in macrophages while no stem cells were present in histological analysis. These results show that while MRI can be used to track cells, the presence of SPIO can lead to false results due to their retention in tissue by other cell types. However, here we use mature DCs that show active migration from the periphery to the popliteal LN. Moreover, the life span of an activated or mature dendritic cell *in vivo*, while somewhat varying according to type and origin, is only a few days and therefore the use of SPIO to MR image DCs over a few days seems appropriate. Furthermore, we predicted that if the signal reduction resulted from free SPIO engulfed by host macrophages or DCs, this signal would have only been observed at the subcapsular region of the LN and not in both the subcapsular sinus and the centre of the popliteal LN as we observed [4], [18].

SPIO labelling of cells together with MRI can offer many advantages. As shown here MRI can reveal that the migration of mature, activated DCs to a LN caused LN growth or swelling. Furthermore, the strongest SPIO effect of hypointensity occurred 24–48 h after DC injection and was similar to previously reported results [4]. A recent publication has suggested that vaccination using SPIO labelled dying tumour cells allowed the imaging of endogenous DCs trafficking following the engulfment of the SPIO labelled tumour cells [18]. The signal in the popliteal LN was first detected day three post-tumour injection and reached a maximum at day 8 [18]. Therefore, in this study, the hypointensity in the popliteal LN was due to the migration of the injected cells and not host DCs that engulf the SPIO labelled DCs and subsequently migrate. In a more recent study, MPIO labelled DCs caused an increased hypointensity for up to 7 days post-injection [9]. However, it was reported that at day 7, there were 50% less fluorescent DCs present in the LN than at day 2. This could be due in part to host DCs and macrophages engulfing and trafficking MPIO to the LN or to the slow clearance of MPIO from the LN after DC death in the LN [9].

The SUMO positive contrast technique together with imaging with varying TEs are sensitive techniques that can be used not only for DC migration but other cell tracking studies using SPIOs. SUMO allows the viewer to see dark pixels in an image due to susceptibility more easily and might also be used to provide quantitative information [19]. Using SPIO labelled DCs it can show the presence intra-nodal as opposed to the supcapsular sinus.

The creation of T_2_^*^ maps from acquisition at different TEs has the potential to be used for quantification of iron *in vivo*. Using the signal intensity at every voxel at different TEs allows a signal decay curve to be used to produce a map that relates to the iron concentration. This was shown in phantom experiments and was also used to confirm compartmentalization of SPIO by DCs (Figure S2). In the *in vivo* DC migration study, however, the creation of a map was difficult because the signal intensity in the popliteal LN at a TE of 4.6 ms had already decayed. The effect of the iron resulted in T_2_^*^ relaxation times too short for accurate measurement in the popliteal LN. This is due to too many SPIO labelled DCs migrating to the LN resulting in strong hypointensity at short TEs. It is possible to acquire MR images at shorter TEs to help produce a T_2_^*^ map, but it is not possible with the high-resolution parameters used in this MR acquisition. However, it would also be possible to titrate the number of SPIO labelled DCs injected so that quantification of DC migration is possible.

In this study we focused on SPIO labelling of DCs and we demonstrated that SPIO had no drastic effect on *in vivo* DC function, including: (i) the growth of popliteal LNs after s.c. DC injection, (ii) the PI and % original dividing cells in the *in vivo* proliferation assay, and (iii) the ability of DCs to protect mice from tumour challenge in the B16-Ova melanoma lung metastases model. Previous reports using an *ex vivo* proliferation assay stated that SPIO labelling of DCs affects the proliferation of T cells [4], [6]. Dekaban et al. showed that the endocytic function was decreased in SPIO labelled DCs when KLH was used as a whole antigen [6]. In the *in vivo* proliferation assay described here, the Ova peptide is used instead of whole Ova protein. This negates the effect of the DCs having to engulf intact antigen. Therefore, the assay does not monitor for the processing and presenting capabilities of DCs. In order to avoid the problem of antigen pulsing, other studies have shown that transfection of DCs to express tumour specific antigens improves DC based tumour vaccination [20].

The B16 murine melanoma tumour model was used to compare the effect of SPIO labelling on DC vaccination capabilities. It should be noted, however, that both the B16 cells and *ex vivo* expanded DCs were cultured in media that contains FBS. Due to this, it is possible that ‘bystander’ immune responses could be elicited by the DCs against components of the FBS that are expressed on B16 tumour cells. This can lead to an anti-tumour effect and could be the reason for the decrease in lung nodules when SPIO labelled DCs not pulsed with Ova_257-264_ were used as a control for DC vaccination. This effect of FBS culture conditions leading to anti-tumour immune responses in the B16 model has been reported in previous studies [21], [22]. Eggert et al. reported that bone marrow derived DCs, either unloaded or tumour-associated antigen loaded, cultured in FBS could protect against a challenge with i.v. or s.c. injected B78-D14, a variant of B16 [21]. However, DCs without FBS in the culture media only led to protection against B78-D14 when they were loaded with the TRP-2 peptide of amino acids 180–188 [21]. Toldbod et al. reported similar results of the antitumour effect of FBS cultured DCs upon B16 challenge that had also been cultured in FBS [22]. In the B16 model described here, the antigen unpulsed DCs resulted in the presence of a range of tumour nodules when challenged with B16 tumour injection and SPIO labelling procedure did not effect the ability of Ova_257-264_ pulsed DCs to completely eliminate Ova_257-264_ expressing tumour cells. However, the possibility remains that uptake of dead SPIO DCs or DC exosomes by lymph node resident DCs, which then act as antigen presenting cells cannot be ruled out.

In conclusion we have demonstrated for the first time a direct comparison of SPIO DCs to their unlabelled counterparts *in vivo*. The conditions described here for SPIO labelling of DCs established an efficient method for the reliable monitoring of DC migration and, in combination with MRI quantification techniques, are the basis for future evaluation of DC immunotherapy in the clinic.

## Supporting Information

Figure S1 **Prussian blue staining of SPIO labelled DCs.** **A**) Unlabelled and **B**) SPIO labelled DCs for 4 h at 37°C and 100 µg/mL of SPIO are fixed and stained with Prussian blue. **C**) Lower magnification image of SPIO labelled DCs.
(TIF)

Figure S2 R_2_, R_2_* and R_2_' maps of MR Image using a 1.5 T clinical MRI scanner. Transverse relaxation rates were used to distinguish intracellular versus free SPIO by the equation R_2_'  =  (R_2_* - R_2_). **A**) R_2_*, **B**) R_2_ and **C**) R_2_' maps. **D**) Values of the maps. Tube numbers 1–7 were free SPIO suspended in gelatin at 2-fold dilutions from 50 µg/mL to 0.78 µg/mL. Tube 8 was gelatin only. Tube numbers 9–13 were of DCs labelled with 100 µg/mL SPIO for 4 h at 1×10^6^ cells/mL and decreasing in 2-fold dilutions. Tube numbers 14-18 were of DCs labelled with 50 µg/mL SPIO for 4 h at 1×10^6^ cells/mL and decreasing in 2-fold dilutions. Tube number 19 is of unlabelled DCs at 1×10^6^ cells/mL.
(TIF)

Figure S3 **DC Phenotype analysis by flow cytometry.** DCs were treated with 100 µg/mL SPIO for 4 h at 37°C and stained for the following phenotypic markers: CD11c, CD40, CD54, CD80, CD86, MHC-I, MHC-II and CCR7, (**A**) – (**H**) respectively. Green line: plus SPIO. Pink line: no SPIO. Solid purple: respective isotype controls. n  =  3.
(TIF)

Figure S4 **Effect of increased TE on MR image.** DCs labelled and unlabelled with SPIO were injected in the right and left legs, respectively. MR images 48 h post-DC injection at TEs of 4.6, 9.2 and 13.8 ms shown in (**A**), (**B**), and (**C**), respectively.
(TIF)

Figure S5 **Prussian blue histology.** 48 h post-SPIO labelled DC injection, popliteal LNs were isolated and snap frozen in OCT. **A**) Prussian blue staining reveals a strong iron stain in the subcapsular sinus. **B**) A zoom image in the center of the image reveals the presence of iron in the center of the LN. **C**) Prussian blue stain of SPIO unlabelled DCs in popliteal LN. **D**) Single cell in popliteal LN after SPIO labelled DC injection.
(TIF)
