# Supplementary figures and images for: Monitoring of In Vivo Function of Superparamagnetic Iron Oxide Labelled Murine Dendritic Cells during Anti-Tumour Vaccination
Source: PLoS One. 2011 May 27;6(5):e19662. doi: 10.1371/journal.pone.0019662 (PMC3103517; doi:10.1371/journal.pone.0019662)

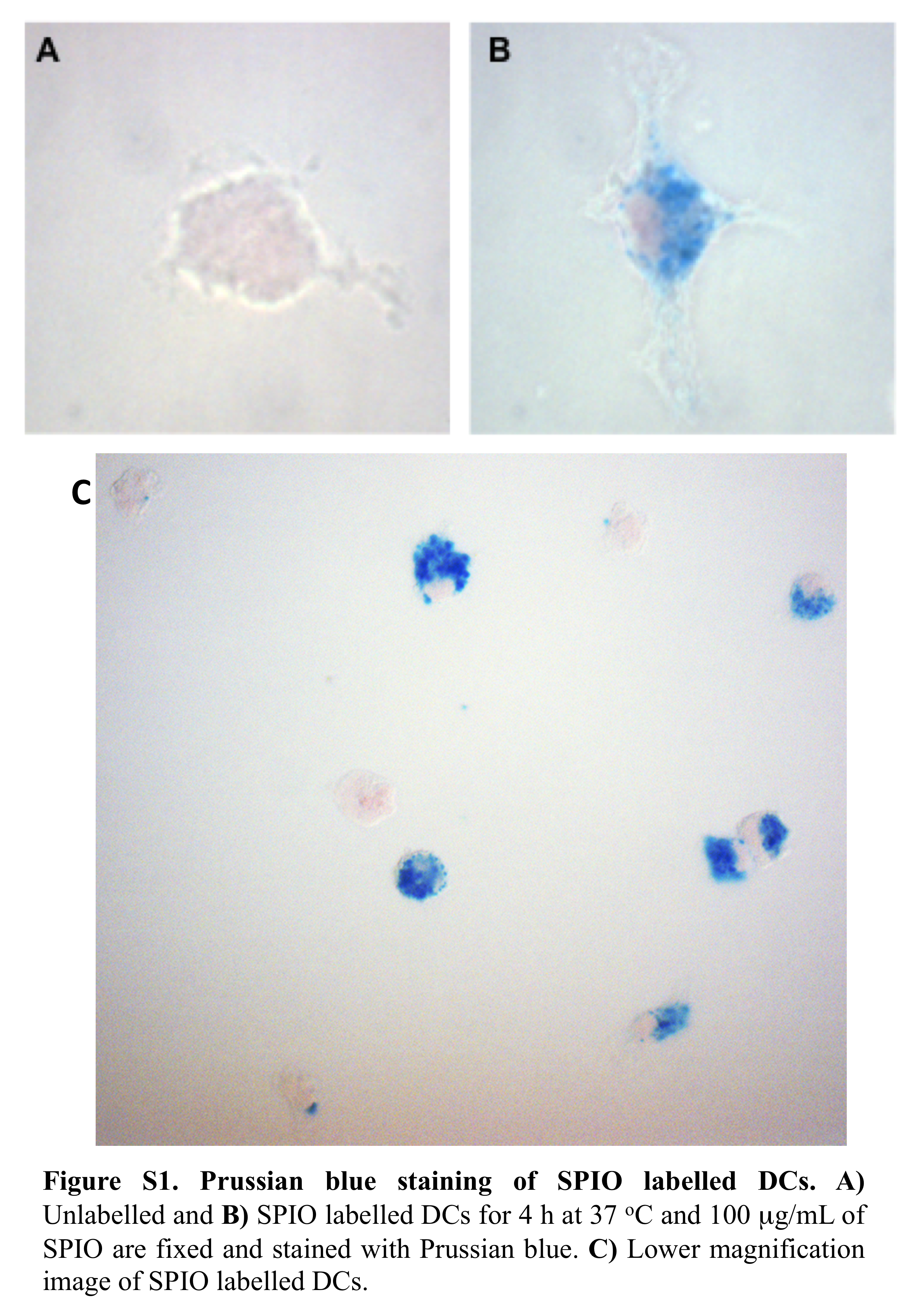

Supplement: Figure S1 — Prussian blue staining of SPIO labelled DCs. A) Unlabelled and B) SPIO labelled DCs for 4 h at 37°C and 100 µg/mL of SPIO are fixed and stained with Prussian blue. C) Lower magnification image of SPIO labelled DCs. (TIF) [file pone.0019662.s001.tif]

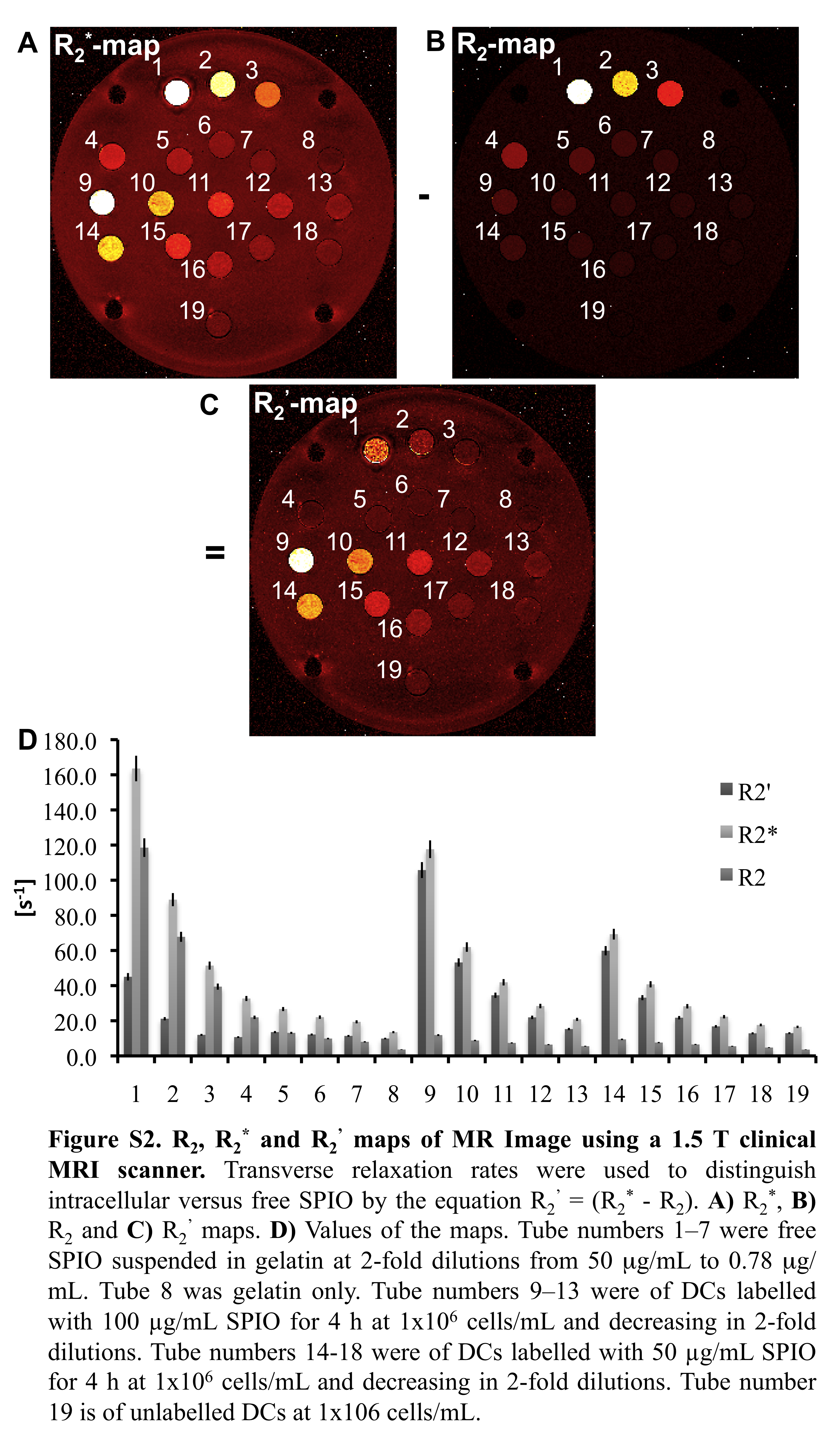

Supplement: Figure S2 — R2, R2* and R2' maps of MR Image using a 1.5 T clinical MRI scanner. Transverse relaxation rates were used to distinguish intracellular versus free SPIO by the equation R2' = (R2* - R2). A) R2*, B) R2 and C) R2' maps. D) Values of the maps. Tube numbers 1–7 were free SPIO suspended in gelatin at 2-fold dilutions from 50 µg/mL to 0.78 µg/mL. Tube 8 was gelatin only. Tube numbers 9–13 were of DCs labelled with 100 µg/mL SPIO for 4 h at 1×10^6^ cells/mL and decreasing in 2-fold dilutions. Tube numbers 14-18 were of DCs labelled with 50 µg/mL SPIO for 4 h at 1×10^6^ cells/mL and decreasing in 2-fold dilutions. Tube number 19 is of unlabelled DCs at 1×10^6^ cells/mL. (TIF) [file pone.0019662.s002.tif]

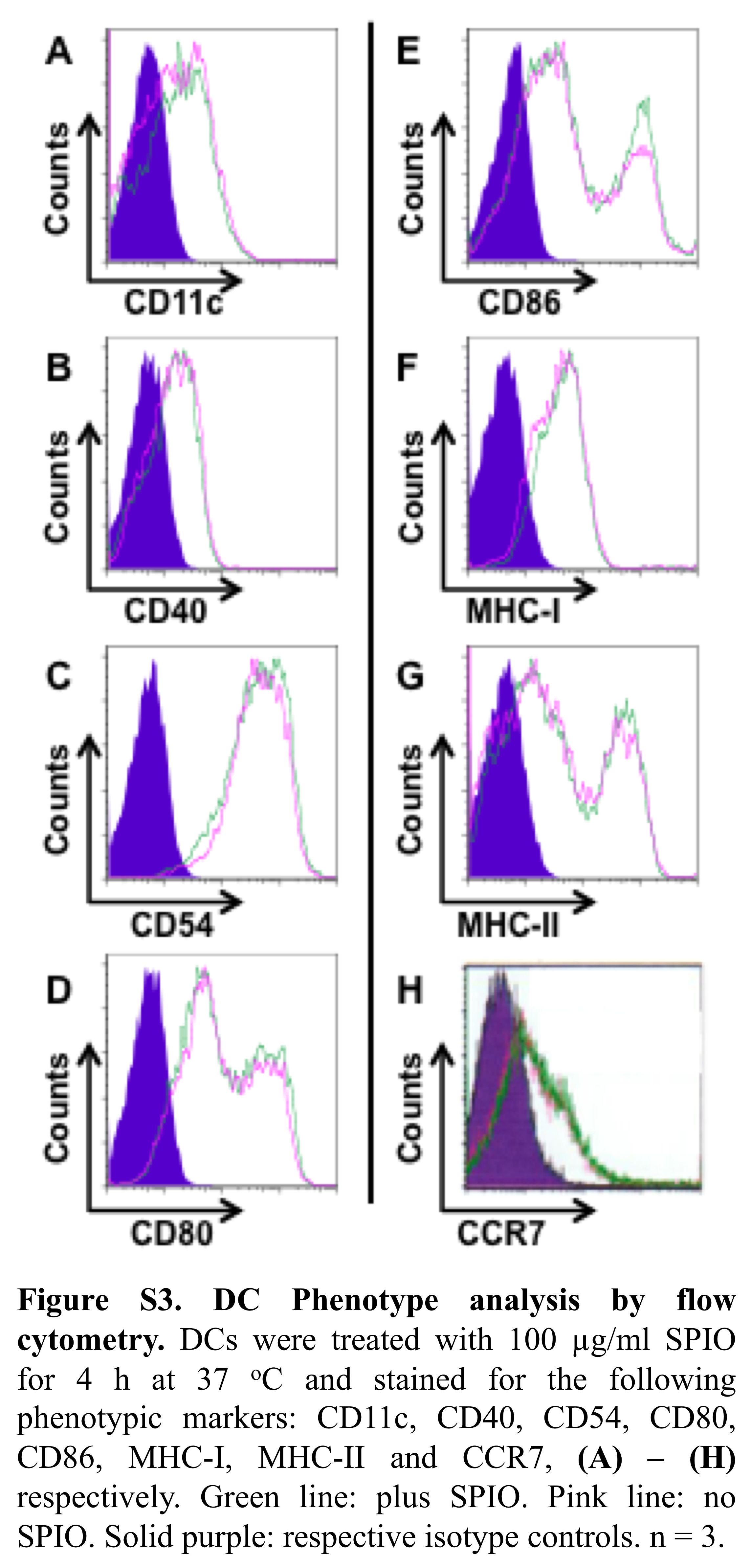

Supplement: Figure S3 — DC Phenotype analysis by flow cytometry. DCs were treated with 100 µg/mL SPIO for 4 h at 37°C and stained for the following phenotypic markers: CD11c, CD40, CD54, CD80, CD86, MHC-I, MHC-II and CCR7, (A) – (H) respectively. Green line: plus SPIO. Pink line: no SPIO. Solid purple: respective isotype controls. n = 3. (TIF) [file pone.0019662.s003.tif]

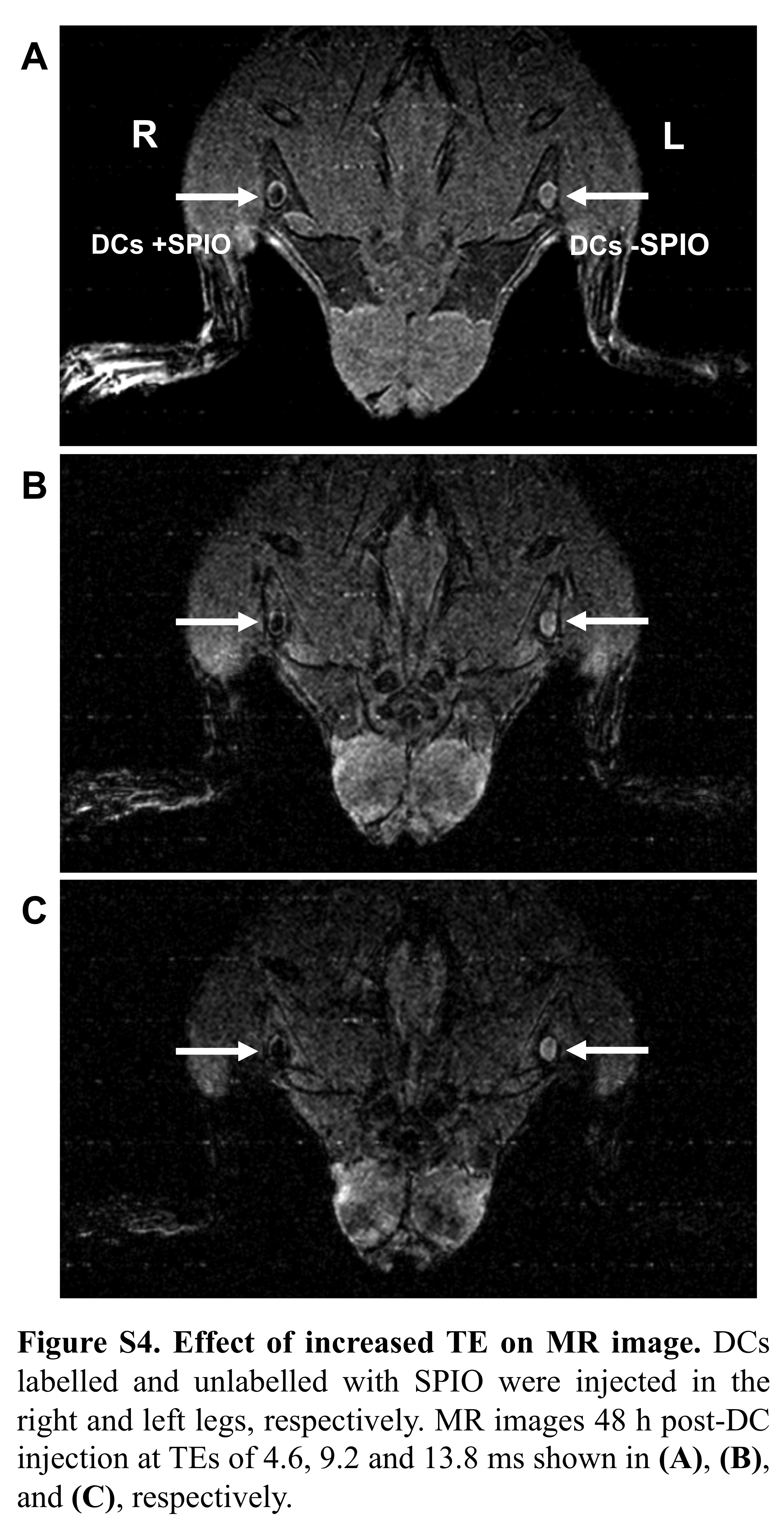

Supplement: Figure S4 — Effect of increased TE on MR image. DCs labelled and unlabelled with SPIO were injected in the right and left legs, respectively. MR images 48 h post-DC injection at TEs of 4.6, 9.2 and 13.8 ms shown in (A), (B), and (C), respectively. (TIF) [file pone.0019662.s004.tif]

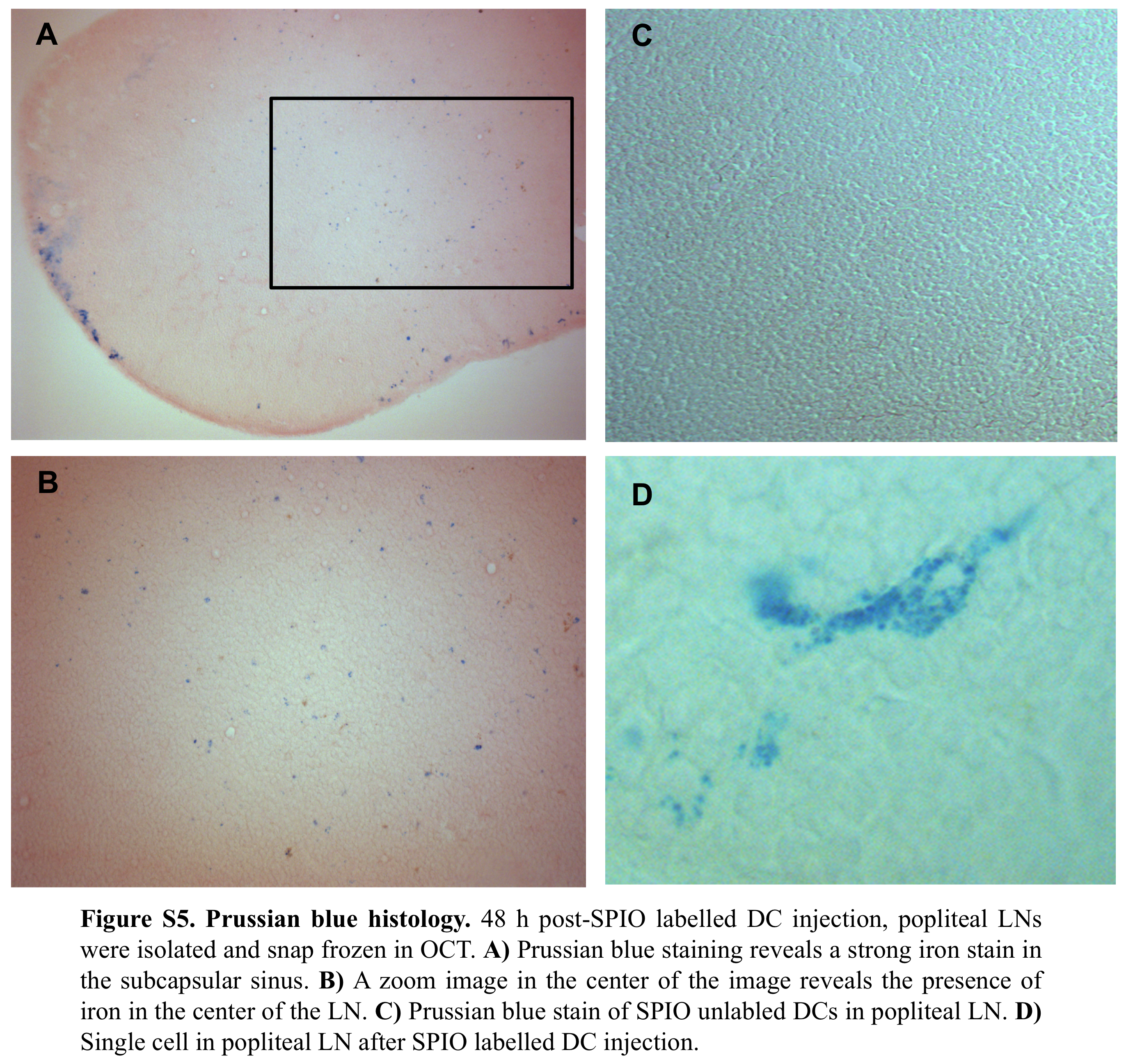

Supplement: Figure S5 — Prussian blue histology. 48 h post-SPIO labelled DC injection, popliteal LNs were isolated and snap frozen in OCT. A) Prussian blue staining reveals a strong iron stain in the subcapsular sinus. B) A zoom image in the center of the image reveals the presence of iron in the center of the LN. C) Prussian blue stain of SPIO unlabelled DCs in popliteal LN. D) Single cell in popliteal LN after SPIO labelled DC injection. (TIF) [file pone.0019662.s005.tif]
